# Categorization Method Affects the Typicality Effect: ERP Evidence from a Category-Inference Task

**DOI:** 10.3389/fpsyg.2016.00184

**Published:** 2016-02-17

**Authors:** Xiaoxi Wang, Yun Tao, Tobias Tempel, Yuan Xu, Siqi Li, Yu Tian, Hong Li

**Affiliations:** ^1^School of Psychology, Southwest UniversityChongqing, China; ^2^Department of Psychology, School of Educational Science and Management, Yunnan Normal UniversityKunming, China; ^3^Key Laboratory of Educational Informatization for Nationalities, Yunnan Normal University, Ministry of EducationKunming, China; ^4^Department of Psychology, University of TrierTrier, Germany; ^5^Research Centre for Brain Function and Psychological Science, Shenzhen UniversityShenzhen, China

**Keywords:** event-related potentials (ERPs), categorization method, typicality effect, category inference, P2, N2, N400

## Abstract

The typicality effect during categorization describes a phenomenon whereby typical items are more easily judged as members of a category than atypical items. Prior studies of the typicality effect have often used an inclusion task, which asks participants to assess whether an item belongs to a category. However, the correct exclusion of non-members is also an important component of effective categorization, which has yet to be directly investigated. Thus, the present study investigated how categorization method (inclusion vs. exclusion) modulates the typicality effect via behavioral and electrophysiological measures. Thirty-two participants (16 in the inclusion and 16 in the exclusion group) were shown six consecutive words that all shared one feature. Then, a seventh word was presented. The inclusion group judged whether the seventh word also possessed the feature, whereas the exclusion group judged whether the word did not possess the feature. The seventh word could be typical, atypical, or a nonmember of the category. Behavioral and event-related potential (ERP) data were collected. Behavioral results showed that the two groups did not differ in accuracy. However, typical items elicited shorter response times than atypical items, and this effect was more pronounced in the inclusion than the exclusion group. With regard to ERPs, interactions between item type and group were shown for the P2, N2, and N400 periods. Within the inclusion group, a typicality effect (indicated by a main effect of item type) was present in the P2 and N400 time windows, while the exclusion group elicited a typicality effect only in the N2 time window. These results provide electrophysiological evidence that an inclusion judgment task is more sensitive to category typicality than is an exclusion task.

## Introduction

The ability to categorize information about the world lies at the core of human knowledge acquisition and experience accumulation. Categories are groups of distinct abstract or concrete items that the cognitive system treats as equivalent for some purpose. Maintaining and using categories involves mental representations that encode key aspects of category members (Murphy and Medin, 1985).

Typicality is one of the most robust properties of a category, reflecting its graded structure. Not every member of a category is considered an equally good exemplar; rather, items lie on a spectrum of category goodness (Rosch and Mervis, 1975). Some items are judged as typical members, whereas others are judged as atypical members. For example, a robin is more often judged to be a typical member of the category bird than is a chicken. The typicality gradient is often thought to reflect the internal membership structure of a concept; that is, a characteristic of one concept, independent of other concepts. Similarly, in category-verification tasks, participants can verify typical items as members of a category more quickly and more accurately than atypical ones (Fujihara et al., 1998).

In addition to behavioral data, typicality effects have been shown in event-related potential (ERP) studies, in which the N400 wave has emerged as an index of typicality effects. Heinze et al. (1998) used word pairs to study typicality effects, in which the first word was the name of a category and the second was the name of a member of that category. There were three conditions: typical member, atypical member, and nonmember. Atypical and nonmember words elicited larger N400 amplitudes than did typical members. The typicality effect is also observed in reasoning tasks (Heinze et al., 1998). Lei et al. (2010) used the ERP technique to study how the typicality of category members affects deductive reasoning. They used a deduction task in which participants first saw a sentence such as “Birds have property X,” followed by a blank screen, after which another item was presented, again followed by a blank screen. Participants were asked to decide if the second item possessed the feature mentioned. N400 mean amplitudes were significantly greater for non-target members than for target members, while words of lower typicality evoked greater N400 amplitudes than words of higher typicality.

As mentioned above, typicality effects have been shown for a range of category-related tasks (Fujihara et al., 1998; Núñez-Pena and Honrubia-Serrano, 2005), but such effects are not inevitable. Fujihara et al. (1998) asked participants to judge whether a word belonged to a given category. Half of the words belonged to the category (target, such as vegetable), and half belonged to a different category (non-target, such as sport). The items were further divided into typical or atypical members of their respective categories. For the non-target category, the typicality effect was found in neither the ERPs nor the reaction times. For the target category, typical words were responded to more quickly than atypical words, and the N400 amplitude was more negative following the atypical words than after the typical words. It seemed that participants were not sensitive to the typicality gradient when items needed to be rejected. Typical items did not show any advantage when they had to be rejected. Thus, the typicality effect can apparently be affected by the categorization method used.

For a long time, typicality was regarded as a similarity gradient of within-category items (Rosch et al., 1976), and inclusion tasks were used to examine the typicality effect. For example, Fujihara et al. (1998) asked participants to judge whether a given conclusion could be classified as the same category as a premise. Lei et al. (2010) used an inference task, asking participants to judge whether a conclusion held the same feature as a premise. Indeed, category tasks as well as inference tasks about inclusion require considerable attention to within-group similarity. The similarity-coverage model is based on the hypothesis that the strength of a categorical argument increases with the degree to which the premise categories are similar to the conclusion category as well as the degree to which the premise categories are similar to the members of the lowest level category that includes both the premise and the conclusion categories (Osherson et al., 1990). Similarly, the feature-based inductive model is based on the hypothesis that argument strength is related to the proportion of the conclusion category's features that are shared by the premise categories (Sloman, 1993). Thus, an inclusion task may be sensitive to the category-typicality gradient. Exclusion is also a common method of category verification Mewhort and Johns (Mewhort and Johns, 2000; Johns and Mewhort, 2002, 2003) argued that, under some circumstances, correct rejections of test items may be made on the basis of the difference between a test item and list items, rather than on the basis of familiarity, as traditional accounts assume. As such, an exclusion task requires more attention toward between-category differences, while the typicality effect is based on category similarity. Thus, exclusion judgments may not be as sensitive to category-typicality effects as are inclusion judgments. Accordingly, there should be distinct cognitive processing and neural mechanisms underlying inclusion and exclusion judgments.

Experimental evidence exists showing that the use of within-group similarity and between-group dissimilarity may lead to different results. Stewart et al. found that classification of a borderline stimulus (similar to atypical items in the present study) was more accurate when preceded by a distant member of the opposite category than when it was preceded by a distant member of the same category. They called this the category-contrast effect (Stewart and Chater, 2002; Stewart and Brown, 2005). The result clearly illustrated that the use of similarity information can be experimentally discriminated from the use of dissimilarity information. When borderline items were compared to members of their own category, similarity information was used. Dissimilarity information was used when making comparisons with distant members of another category. Between-group differences may be more obvious, so it will be easier to make such judgments.

It has been suggested that the frontally distributed P2 component is linked with exogenous, stimulus driven attention (Freunberger et al., 2007). P2 components may indicate relatively early sensory stages of information processing (Thomas et al., 2007). In a prior study using a partially congruent categorization task, P2 amplitudes were found to be lower when more cognitive control was needed (Chen et al., 2008). Recent research has also shown reduced P2 amplitudes with enhanced emotional control skills, as measured by a social skills inventory (Meaux et al., 2014), suggesting that adults exhibited larger anterior distributed P2 amplitudes in a two-choice oddball tasks compared to adolescents. Since adolescents need more effort to perform the task, this result indicates that more control leads to lower P2 (Yuan et al., 2015). Therefore, P2 may relate to the strength of cognitive or emotional control in a task. Frontal to central N2 in behavioral inhibitory control tasks has been accepted as an index for response conflict monitoring, and for increased intentional engagement that forms the basis for the subsequent response inhibition (Eimer, 1993; Van Veen and Carter, 2002; Yuan et al., 2008a; Yu et al., 2009). With regard to the present study, we assume exclusion judgments to be associated with experiencing greater conflict than inclusion judgments. When performing an exclusion or inclusion judgment, participants first categorize the items and then make their inferences. In exclusion judgments, objects responded to with a “No” in category processing should receive a “Yes” response in the inference stage. In contrast, in inclusion judgments, the answers are coordinated in these two stages. Therefore, exclusion judgments may elicit larger N2 waves than inclusion judgments.

In summary, inclusion and exclusion are both effective approaches to categorization, which rely on within-group similarity and between-group differences, respectively. The present study focused on the typicality effect and how it differs for exclusion vs. inclusion judgments. Regarding the electrophysiological correlates of the typicality effect under inclusive and exclusive categorization, we expected the typicality effect indexed by the N400 component to differ between an exclusion and inclusion group. The inclusion group was expected to be more sensitive to the typicality effect, which is shown by the effect of item type in the N400 time window. Moreover, differences in cognitive processing of inclusion and exclusion were anticipated in early ERP components, including P2 and N2; specifically, lower P2 and larger N2 were expected for the exclusion group.

## Materials and methods

### Participants

Eighty healthy undergraduate students rated the experimental materials in a pilot study. Another 32 healthy undergraduate students participated in the main study. All participants (age range: 18–22 years), both in the pilot and the main study, were right-handed with normal or corrected-to-normal vision, and they all received reimbursement for their participation. The 32 participants in the main study were divided equally into the two groups: Nine women and seven men each in the inclusion group (mean age: 20.19, *SD* = 1.36), and exclusion group (mean age: 19.81, *SD* = 1.27). No differences of age were discovered in the independent *t*-test, *t*_2−*tailed*_(30) = 0.814, *p* = 0.422. The study was approved by the Local Review Board for Human Participant Research, and written informed consent was obtained from all participants. The experiment was conducted in accordance with the ethical principles of the 1964 Declaration of Helsinki (World Medical Organization, 1996).

### Materials

Sixteen categories were chosen: 4 animals (mammal, fish, bird, and insect), 4 plants (fruit, vegetable, flower, and traditional Chinese medicine), 4 concrete objects (clothes, tools, musical instruments, and furniture), and 4 abstract categories (colors, personality traits, emotions, and classifiers). A classifier is a grammar system of nominal classification (Senft, 2000). In Chinese, classifier systems are usually nouns organized around semantic features such as animacy, shape, function, size, rigidity, and social importance (Saalbach and Imai, 2007). In the pilot study, 40 participants were asked to name as many members of these 16 categories as possible. Another 40 participants rated the typicality of the generated members on a 7-point scale, where 7 indicated *typical*, 4 indicated *uncertain*, and 1 indicated *atypical*. For each category, 5 atypical words and 12 typical words were selected for the main study. The typical words (*M* = 6.51, *SD* = 0.22) differed significantly from the atypical words [*M* = 4.65, *SD* = 0.55; *t*_(1, 270)_ = −40.991, *p* < 0.01, *d* = 5.54] in terms of typicality. In addition, 160 exemplars of categories unrelated to the experimental categories were chosen as filler items.

### Procedure

The experiment consisted of learning and test phases. There were two parts to the learning phase; namely, category learning followed by classification. In each trial of the classification test, item and category names were simultaneously presented on the screen, and the participant had to judge whether the item was a member of the category. Altogether, 272 items had to be classified. Only when accuracy reached 90% did the participant proceed with the next stage. If this threshold was not reached, the participants repeated category learning and classification.

In the test phase, the participants were divided into one of two judgment groups: inclusion or exclusion. There were 320 trials in total. For each of the 16 categories there were 20 trials, which consisted of 5 typical endings, 5 atypical endings, and 10 nonmember endings. The proportions of member and nonmember trials were equal. A trial consisted of the sequential presentation of words with a judgment required regarding the last word. A fixation cross was presented in the center of a gray screen for 500 ms at the beginning of each trial. Subsequently, a series of six words appeared in the center of the screen in random order. Each word was presented for 1000 ms, followed by a blank screen for 500 ms. These six words all belonged to the same experimental category and were randomly drawn from the 12 selected typical exemplars. Subsequently, participants judged the category membership of the seventh word. Depending on the experimental group, the participants were instructed to assess whether the word was included within or excluded from the category of the previously presented exemplars. In the inclusion group, the instructions were: “You will see a series of six words. These six words all are members of the same category. All members of this category possess a feature X, whereas nonmembers of this category do not possess this feature. After these six words, you will see a seventh word. Your task is to judge whether the seventh word possesses the feature X. If you are uncertain about the answer, press the *NO* button.” In the exclusion group, the instructions were: “You will see a series of six words. These six words all are members of the same category. All members of this category possess a feature X, whereas nonmembers of this category do not possess this feature. After these six words you will see the seventh word. Your task is to judge whether the seventh word DOES NOT possess the feature X. If you are uncertain about the answer, press the *NO* button.” Participants used an SR-BOX (Psychology Software Tools, Inc., Sharpsburg, USA) to respond. The keys *1* and *5* of the SR-BOX were labeled with *Yes* and *No*.

### ERP recordings and data analysis

Brain electrical activity was recorded from 64 Ag/AgCl electrodes mounted on an elastic cap (Brain Products, Munich, Germany), with a ground electrode on the medial frontal line, using the FCz online reference and offline algebraic re-reference analysis to the left and right mastoids (Luck, 2014). The impedance of all electrodes was maintained below 10 kΩ. The vertical electrooculograms (EOGs) were recorded supra-and infra-orbitally at the left eye. The horizontal EOG was recorded from the left vs. right orbital rim. The signals were amplified using a recording band-pass of 0.05–100 Hz (FIR filter,) and continuously sampled at 500 Hz/channel for offline analysis. ERPs was computed off-line using the Vision Analyzer software developed by the Brain Products Company (Munich, Germany). EEG was band-pass filtered from 0.1 to 24 Hz for offline analysis (The IIR Filters transform applied to filter EEG data. The filters are implemented as phase shift-free Butterworth filters, 24 dB/octave), and baseline corrected. EOG artifacts (blinks and eye movements) were corrected using the eye movement correction algorithm recommended by Gratton et al. (1983). Offline computerized artifact rejection was used to eliminate trials with EOG artifacts (mean EOG voltage exceeding ±80 μV). Additionally, trials with artifacts due to amplifier blocking or muscle potentials were eliminated, as were trials with peak-to-peak deflections exceeding ±80 μV. Only the ERPs elicited by the seventh word were examined. Epochs of 1200 ms, time locked to the seventh word, including a 200 ms prestimulus baseline, were extracted from the ongoing EEG, segmented, and averaged.

Repeated measures ANOVAs were performed on the mean amplitude of the time windows, with item type (three levels: typical, atypical, nonmember), laterality (three levels: left, middle, and right sites), and frontality (five levels: frontal: left—F3, middle—Fz,right—F4; frontal central: left—FC3, middle—FCz, right—FC4; central: left—C3, middle—Cz, right—C4; central parietal: left—CP3, middle—CPz, right—CP4; parietal: left—P3, middle—Pz, right—P4) as repeated factors and group (two levels: inclusion, exclusion) as between subject factors. For all analyses, *p*-values were corrected using the Greenhouse Geisser method.

## Results

### Behavioral responses

**Figure 1:**
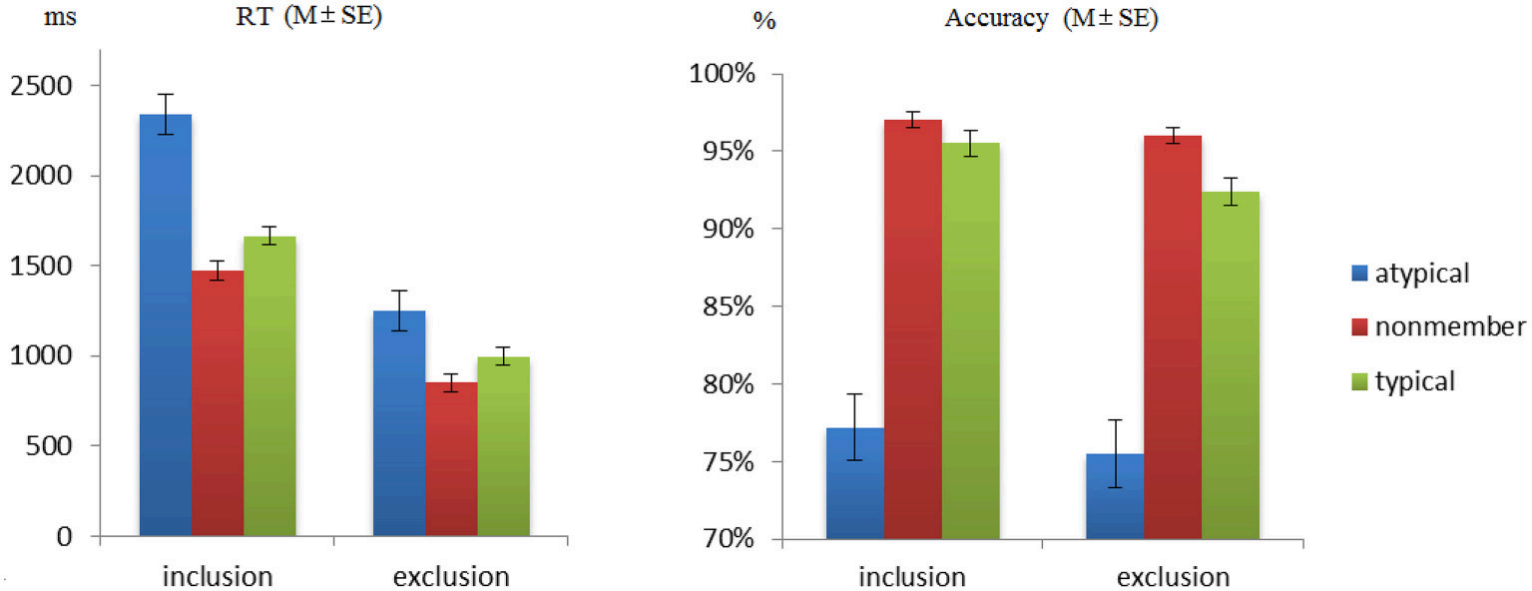
**The mean reaction time for exclusion group was significantly shorter than which of inclusion group, typicality effect was only found within inclusion group**. The accuracy showed similar pattern in these two groups.

A 2 (group: inclusion group, exclusion group) × 3 (item type: typical, atypical, nonmember) ANOVA with repeated measures on the second factor examined accuracy in the test phase (see Figure 1). The main effect of item type was significant, *F*_(2, 60)_ = 128.506, *p* < 0.001, $\eta_{\text{p}}^{2}=0.711$. *Post-hoc* tests with Bonferroni correction showed that the accuracy for nonmembers was significantly higher than that for both typical members (*p* = 0.004) and atypical members (*p* < 0.001). In addition, typical members' accuracies were significantly higher than those of the atypical members (*p* < 0.001). The main effect of group was not significant, *F*_(1, 30)_ = 3.004, *p* = 0.093, η${}_{\text{p}}^{2}=0.091$, nor was the interaction, *F*_(2, 60)_ = 0.319, *p* = 0.637, η${}_{\text{p}}^{2}=0.011$.

A similar 2 × 3 ANOVA examined reaction times (RTs) in the test phase. The interaction between group and item type was significant, *F*_(2, 60)_ = 7.686, *p* = 0.006, η${}_{\text{p}}^{2}=0.204$. The main effect of item type was significant, *F*_(2, 60)_ = 50.701, *p* < 0.001, η${}_{\text{p}}^{2}=0.628$, whereby the exclusion group reacted faster than the inclusion group, *F*_(1, 30)_ = 109.249, *p* < 0.001, η${}_{\text{p}}^{2}=0.785$. Simple effect analyses found a significant effect of item type both in the inclusion, *F*_(2, 30)_ = 28.380, *p* < 0.001, η${}_{\text{p}}^{2}=0.654$, and exclusion groups, *F*_(2, 30)_ = 34.078, *p* < 0.001, η${}_{\text{p}}^{2}=0.694$. In both groups, the same pattern of differences was observed across the three conditions. That is, nonmember items were associated with faster RTs than typical (inclusion: *p* = 0.024, exclusion: *p* = 0.016) and atypical items (inclusion: *p* < 0.01, exclusion: *p* < 0.01), and typical items elicited faster RTs than atypical items (inclusion: *p* < 0.01, exclusion: *p* < 0.01). To further test how the typicality effect changed by categorization method, the RT difference for typical minus atypical items was employed as an index of the typicality effect. An independent *t*-test showed that the size of the typicality effect was significantly larger in the inclusion group than in the exclusion group, *t*_(1, 30)_ = 3.293, *p* = 0.004, *d* = 1.166.

### Event-related potentials

**Figure 2:**
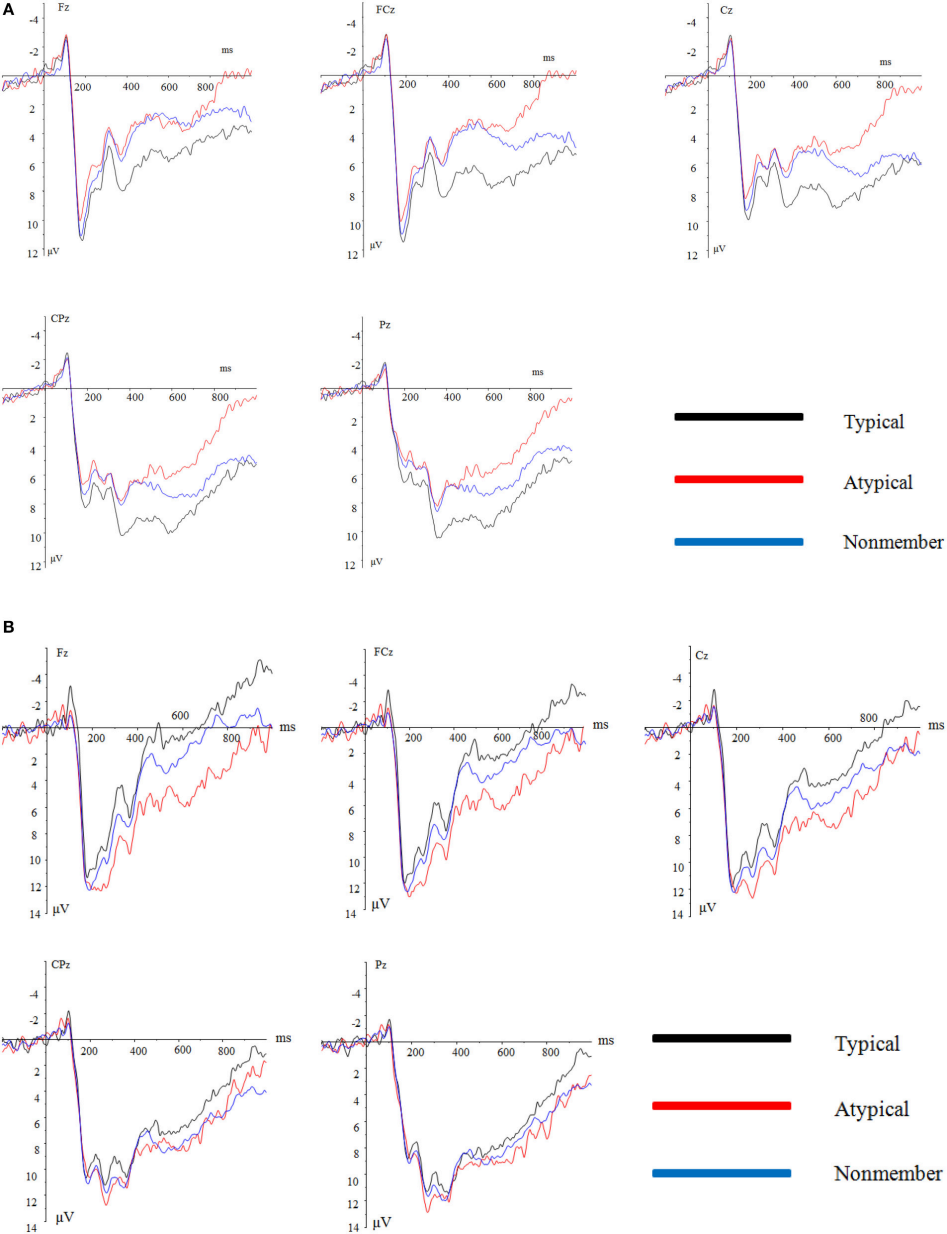
**(A)** Grand average ERPs at Fz, FCz, Cz, CPz, and Pz for the typical, atypical, and nonmember items in the inclusion group. **(B)** Grand average ERPs at Fz, FCz, Cz, CPz, and Pz for the typical, atypical, and nonmember items in the exclusion group.

**Figure 3:**
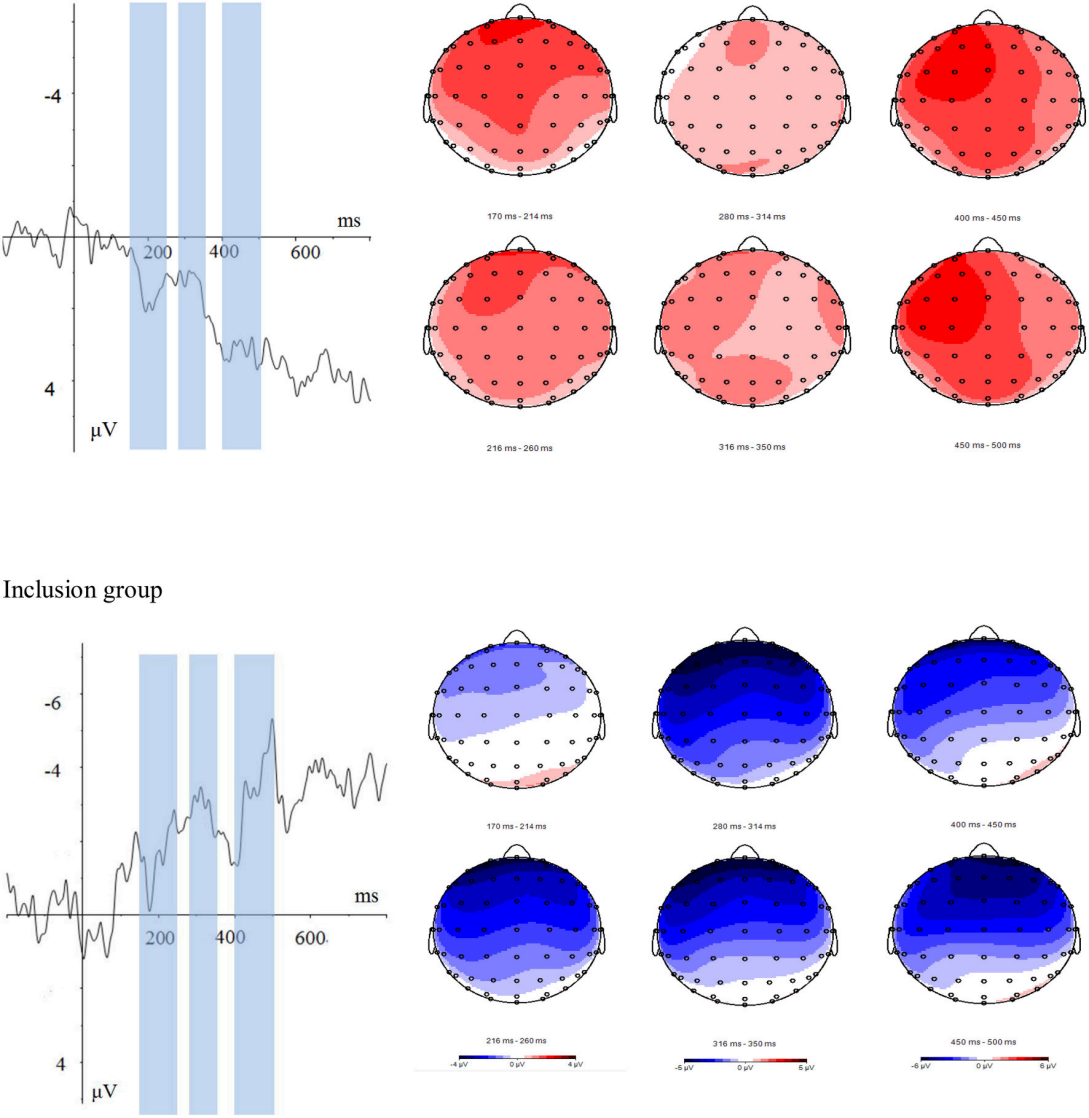
**Typical-atypical ERP differences at FCz, and the topographical distribution of the voltage amplitudes of typical-atypical ERP differences in P2 (170–260 ms), N2 (280–350 ms), and N400 (400–500 ms) time intervals in the inclusion (top panel) and exclusion (bottom panel) groups**. The time windows are highlighted with blue bars. The figure illustrated a sustained effect which runs in opposite directions for inclusion and exclusion condition.

The averaged ERPs and topographical maps (see Figures 2, 3) showed that the amplitude difference across three item type condition in both groups started at about 170 ms post stimulus. Time window for analyzing were found as positive peak between 170 and 260 ms (P2), negative peaks between 280 and 350 ms (N2), and between 400 and 500 ms (N400). However, the pattern of the waves differed between the inclusion and exclusion groups. We analyzed ERPs in each interval using separate 3 (electrode laterality: left, middle, right) × 5 (electrode frontality: frontal, frontal central, central, parietal central, parietal) × 3 (item type) × 2 (group) ANOVAs with repeated measures on the first three factors.

#### 170–260 ms interval

The main effect of item type was not significant, *F*_(2, 60)_ = 0.156, *p* = 0.809, $\eta_{\text{p}}^{2}$ = 0.005, whereas the main effect of group was, *F*_(1, 30)_ = 8.273*, p* = 0.007, $\eta_{\text{p}}^{2}=0.216$. The exclusion group generally elicited a larger positive wave than the inclusion group. More importantly, the interaction between item type and group was significant, *F*_(2, 60)_ = 4.753, *p* = 0.019, $\eta_{\text{p}}^{2}$ = 0.137. Simple effects tests showed that the effect of item type was not significant in the exclusion group, *F*_(2, 30)_ = 1.345, *p* = 0.271, $\eta_{\text{p}}^{2}$ = 0.082, while significant differences were present in the inclusion group, *F*_(2, 30)_ = 6.080, *p* = 0.012, η^2^ = 0.288. *Post-hoc* tests (Bonferroni) showed that atypical items elicited lower amplitude positive waves than did the typical items (*p* = 0.04). No other differences were found.

The main effects of frontality *F*_(4, 120)_ = 26.73, *p* < 0.001, η${}_{\text{p}}^{2}$ = 0.47 and laterality *F*_(2, 60)_ = 16.85, *p* < 0.001, η${}_{\text{p}}^{2}=0.36$ were significant. *Post-hoc* tests of frontality indicated that the P2 amplitudes at the frontal site were more positive than at the central site (*p* = 0.032), central parietal site (*p* < 0.001), and parietal site (*p* < 0.001). P2 amplitudes at the frontal central site were more positive than those at the central (*p* < 0.001), the central parietal (*p* < 0.001), and parietal sites (*p* < 0.001). P2 amplitudes at the central site were more positive than those at the central parietal (*p* < 0.001) and parietal sites (*p* < 0.001). Furthermore, the P2 amplitudes at the central parietal site were more positive than that at the parietal site (*p* = 0.004). *Post-hoc* tests of laterality indicated that the P2 amplitudes at the middle site were more positive than those at either the left (*p* =0.001) or the right sites (*p* < 0.001). There was no significant difference between the left and right sites (*p* = 0.061).

The three-way interaction of item type, frontality, and group was significant *F*_(8, 240)_ = 4.307, *p* < 0.01, $\eta_{\text{p}}^{2}=0.126$. Further analysis showed that item type and frontality interactions were not found in the inclusion group, *F*_(8, 120)_ = 1.132, *p* = 0.347, η${}_{\text{p}}^{2}$ = 0.07, while the interactions were found in the exclusion group *F*_(8, 120)_ = 3.83 *p* = 0.024, η${}_{\text{p}}^{2}$ = 0.203. Item type showed difference modes in different lines, as is detailed in Table 1.

**Table 1:** **Typicality effect demonstrated for each line of the exclusion group (*p* value of *post-hoc* test Bonferroni)**.

	**Frontal**	**Frontal central**	**Central**	**Central parietal**	**Parietal**
Typical-atypical	0.027	0.068	0.421	0.718	1.00
Typical-nonmember	0.121	0.148	0.918	0.883	1.00
Atypical-nonmember	1.00	1.00	1.00	1.00	1.00

*In addition, there were no other main effects or interactions (all p > 0.154), as detailed in Appendix Table 1 (Supplementary Material)*.

#### 280–350 ms interval

The main effect of group was significant, *F*_(1, 30)_ = 5.629, *p* = 0.024, η${}_{\text{p}}^{2}$ = 0.158, whereby the exclusion group showed a larger amplitude negative wave than the inclusion group. The main effect of item type was not significant, *F*_(2, 60)_ = 1.252, *p* = 0.290, η${}_{\text{p}}^{2}$ = 0.004, There was also a significant item type by group interaction, *F*_(2, 60)_ = 5.260, *p* = 0.012, η${}_{\text{p}}^{2}=0.149$. Simple effects tests showed no significant effect of item type in the inclusion group, *F*_(2, 30)_ = 1.933, *p* = 0.170, η${}_{\text{p}}^{2}=0.114$, but a significant effect in the exclusion group, *F*_(2, 30)_ = 3.712, *p* = 0.036, η${}_{\text{p}}^{2}=0.198$. In the exclusion group, typical items elicited larger negative amplitudes than atypical (*p* = 0.008) but were no difference from nonmembers (*p* = 0.452), whereas there was no significant difference between atypical and nonmember items (*p* = 0.861).

The main effect of frontality reached marginal significance, *F*_(4, 120)_ = 3.737, *p* = 0.052, η${}_{\text{p}}^{2}$ = 0.111, while the main effect of laterality *F*_(2, 60)_ = 0.180, *p* = 0.833, η${}_{\text{p}}^{2}=0.006$ did not. *Post-hoc* tests of frontality did not find a significant difference between either of the two lines (all *p* > 0.09). Significant interactions were found between item type and frontality *F*_(8, 240)_ = 4.335, *p* = 0.01, η${}_{\text{p}}^{2}=0.126$, as well as between frontality and laterality *F*_(8, 240)_ = 4.101, *p* = 0.002, η${}_{\text{p}}^{2}$ = 0.120. More importantly, a three-way interaction was found in item type, frontality, and group, $F_{\left(8,240\right)}=3.471,p=0.026,\eta_{\text{p}}^{2}=0.104$. Further analyses showed that the interaction of item type and frontality was not significant in the inclusion group, *F*_(8, 120)_ = 0.399, *p* = 0.754, whereas it was in the exclusion group *F*_(8, 120)_ = 6.302 *p* = 0.004, η${}_{\text{p}}^{2}$ = 0.296. Simple effects of item type were significant at frontal (*p* = 0.005), frontal central (*p* = 0.004), central (*p* = 0.004), and parietal central (*p* = 0.016) but not parietal sites (*p* = 0.081). There were no other main effect or interaction found (all *p* > 0.201), detailed see Appendix Table 2 (Supplementary Material).

#### 400–500 ms interval

This time interval was chosen to quantify the N400 component. Neither the main effects of item type, $F_{\left(2,60\right)}=1.010,p=0.361,\eta_{\text{p}}^{2}=0.033$, or group, *F*_(1, 30)_ = 0.381, *p* = 0.542, $\eta_{\text{p}}^{2}=0.013$, were significant. The interaction between item type and group was significant, *F*_(2, 60)_ = 6.476, *p* = 0.005, η${}_{\text{p}}^{2}=0.178$. Simple effects tests showed that the effect of item type was significant in the inclusion group, *F*_(2, 30)_ = 7.753, *p* = 0.004, η${}_{\text{p}}^{2}=0.341$, but not in the exclusion group, *F*_(2, 30)_ = 2.355, *p* = 0.135, η${}_{\text{p}}^{2}=0.136$. *Post-hoc* tests with Bonferroni correction in the inclusion group showed that both atypical items (*p* = 0.018) and nonmembers (*p* = 0.044) showed larger N400 amplitudes than did the typical items, whereas no difference between the atypical items and nonmembers was found (*p* = 0.833).

The main effects of frontality *F*_(4, 120)_ = 16.517, *p* < 0.001, η${}_{\text{p}}^{2}$ = 0.355, and laterality *F*_(2, 60)_ = 5.528, *p* = 0.007, η${}_{\text{p}}^{2}=0.156$, were significant. *Post-hoc* tests of frontality indicated that the N400 amplitudes at the frontal site were more negative than those at the central parietal (*p* = 0.003) and parietal sites (*p* = 0.001). The frontal central site amplitudes were more negative than those at the central parietal (*p* = 0.003) and parietal sites (*p* = 0.001), and the central site amplitudes were more negative than those at the central parietal (*p* = 0.001) and parietal sites (*p* = 0.002). *Post-hoc* tests of laterality indicated that the negative amplitudes at the left sites were larger than those at the middle (*p* = 0.009) and right sites (*p* = 0.040). No other main effects or interactions were significant (all *p* > 0.066), detailed see Appendix Table 3 (Supplementary Material).

#### Timing of typicality effects for the inclusion and exclusion groups

The above results imply that the typicality effect occurred earlier for the inclusion (P2, 170–260 ms) than the exclusion group (280–350 ms). In order to test the reliability of this timing effect, we conducted a separate ANOVA with timing (2 levels: N2, P3), typicality effect, and group as factors for electrode FC3, since this electrode showed the largest main effect in both groups. There were significant effects of timing, *F*_(1, 30)_ = 15.720, *p* < 0.001, η${}_{\text{p}}^{2}=0.344$ and group *F*_(1, 30)_ = 6.929, *p* = 0.013, η${}_{\text{p}}^{2}$ = 0.188. Most importantly, a three-way interaction, *F*_(1, 30)_ = 15.232, *p* < 0.001, η${}_{\text{p}}^{2}=0.338$, was observed. Simple effects analysis illustrated a typicality effect in the exclusion group in both the P2, *F*_(1, 15)_ = 5.240, *p* = 0.037, η${}_{\text{p}}^{2}$ = 0.259, and N2, *F*_(1, 15)_ = 8.405, *p* = 0.011, η${}_{\text{p}}^{2}$ = 0.359, time windows. In the exclusion group, a typicality effect was not present in the P2 time window, *F*_(1, 15)_ = 0.793, *p* = 0.387, η${}_{\text{p}}^{2}=0.050$, but it was evident in the N2 time window, *F*_(1, 15)_ = 15.260, *p* = 0.001, η${}_{\text{p}}^{2}=0.504$. These results confirm that the inclusion group elicited a typicality effect earlier than did the exclusion group.

## Discussion

### Behavioral data

The primary aim of the present study was to examine whether methods of reasoning affect the typicality effect. Accuracy did not differ between the inclusion and exclusion groups, but response times did. That there was no difference in accuracy between the two methods indicates that both inclusion and exclusion are equally effective in classifying items.

The inclusion group showed slower reaction times than did the exclusion group, which indicates different cognitive processes underlying inclusion and exclusion judgments. Exclusion judgments require attention toward differences between categories, whereas inclusion judgments require more attention toward similarity within categories. Differences between categories should be clearer than within-group similarities. This explains why the exclusion group reacted faster than the inclusion group. Kittur et al. (2006) proposed a two-stage model of categorization, assuming that items are first judged for category membership and then graded within the category. Based on this model, classification and typicality judgments may occur in different processing periods. In an inclusion task, both stages must be completed, whereas an exclusion task requires only completion of the first stage. This may explain the faster RTs in the exclusion group.

The interaction between group and item type showed that differences in RTs between typical and atypical members were larger in the inclusion than the exclusion group, which suggests that the inclusion task required more precise processing than did the exclusion task. Furthermore, it was sensitive to category typicality. In addition, RTs to nonmembers were the fastest. This result is consistent with that of a previous study by Lei et al. (2010), who found that rejection of nonmembers had the fastest RTs in a deductive-reasoning task. In summary, both inclusion and exclusion are effective methods of categorization, but they are based on different cognitive processes.

### ERP data

The N400 component has been regarded as an index of typicality in previous studies (Fujihara et al., 1998; Ellis and Nelson, 1999), but evidence has shown the typicality effect can be observed in earlier time windows, such as N1 and P2 (Lei et al., 2010). In the present study, within the 170–260 ms time window, obvious central-frontal P2 activity was elicited for all three item types in both the inclusion and exclusion groups. Frontal P2 activation within 200 ms of stimulus onset is indicative of rapid detection of typical stimulus features and is assumed to relate to perceptual processing (Thorpe et al., 1996; Yuan et al., 2007). The interaction between item type and group in this stage indicates different perceptual patterns between these two groups. The main effect in the inclusion group demonstrated that participants were sensitive to typicality during perceptual processing. Although, atypical items were members of the category, they were dissimilar to the mental representation of the typical item, so they were quickly regarded as not belonging to the category. Furthermore, the generally lower-amplitude P2 wave of the inclusion group may indicate its sensitivity to feature-detection processes. Similarly, in emotional sensitivity studies, participants elicit lower P2 when presented with negative, rather than positive, materials, indicating that people are sensitive to the negative emotional attentional bias (Yuan et al., 2007, 2015). Whereas exclusion judgment relies on between-groups differences, feature detection for this group was easier than for the inclusion group, as shown in the faster reaction times and equivalent accuracy of the exclusion group. No typicality effect was found.

In the present study, participants were shown six typical words belonging to a certain category before they saw the seventh one. The first six words were supposed to lead to the activation of the typical member of the category (Núñez-Pena and Honrubia-Serrano, 2005). Within the inclusion group, participants needed to find something common in the first six words. Atypical members are more ambiguous than both typical and nonmembers because they are just partially like the typical ones, so they are most difficult to judge among the three item types, that is, more cognitive control is needed. In accordance with the greater control in regards to atypical members, lower P2 amplitudes were elicited, and this was in line with previous investigations of partially incongruent categorization (Chen et al., 2008). However, the exclusion task was easier than the inclusion task, so larger P2 amplitudes were observed in the exclusion group.

N2 indicates conscious processing of information (Sergent et al., 2005) and may also reflect the appearance of conflict (Yeung and Cohen, 2006), and the increased attentional engagement that forms the basis for subsequent response inhibition (Van Veen and Carter, 2002; Yuan et al., 2008b; Yu et al., 2009). An interaction between item type and group was observed in the N2 window, and a main effect of item type was present only in the exclusion group. Within the exclusion group, typical items showed a larger negative deflection in N2 than did atypical items, which suggests that typical items in the exclusion group may cause relatively more conflict. The conflict may be caused by category membership and experimental task requirements. The present task included two stages of cognitive processing, first classifying whether the item belonged to the former category, followed by the inference task. Within the exclusion group, the category membership activated in Stage One should have been inhibited in Stage Two, as items accepted in the classification stage should be rejected in the reasoning stage; that is, more cognitive control was needed. More specifically, the N2 wave found in the present study should be regarded as an N2b component, as N2b was larger in response to nontargets as opposed to targets, even though it was elicited by both with a frontal or central scalp distribution (Pritchard et al., 1991; Folstein and Van Petten, 2008). In the present study, main effects were found in the exclusion group during the N2 time window, and the typical item that needed to be rejected elicited the largest N2 and showed a central to frontal distribution, which matches the N2b criterion. However, for the inclusion group, the items accepted in the classification stage should also have been accepted in the reasoning stage. Therefore, there was not as strong a conflict in this group as there was in the exclusion group, and an item-type main effect was not elicited. Additionally, the exclusion group showed a generally higher-amplitude N2 wave than the inclusion group, which suggests that more inhibition control was needed in the exclusion group during the N2 time window.

More importantly, we observed a significant item-type effect in both P2 and N2 amplitudes for the inclusion group, while the exclusion group only showed this effect for N2 amplitude, as indicated by our timing analysis. This suggests that inclusion and exclusion judgments are associated with different mechanisms of typicality processing. That is, inclusion judgments are more sensitive to typicality information than exclusion judgments, which may account for the earlier typicality effect during the inclusion conditions.

The N400 component has been observed in most ERP studies of category typicality processes and is regarded as an index of the typicality effect (Fujihara et al., 1998; Lei et al., 2010; Kutas and Federmeier, 2011). In the present study, the typicality effect was observed in the inclusion group, consistent with prior studies (Fujihara et al., 1998; Núñez-Pena and Honrubia-Serrano, 2005), but it was not seen in the exclusion group. The paradigm used in the present study was similar to that of Núñez-Pena and Honrubia-Serrano (2005). The only difference is that the prior study only required participants to complete a classification task, while an inference task was used in the current study. Our results for the inclusion group are consistent with those of Nunez-Pena and Honrubia-Serrano, but we did not observe a typicality effect in the exclusion group in the N400 time window. For exclusion judgments, participants just need to determine non-membership. Accordingly, attention should be paid to between-group differences, for which typicality is not important. Furthermore, the present task actually consisted of two stages; namely, classification and reasoning. The N400 component is regarded as an index of the typicality effect in categorization and reasoning studies. The N400 component in the inclusion group may index typicality during both classification and reasoning, wherein the classification of atypical items requires cognitive inhibition of the distracting, conflicting perceptual representations. This process has been established to elicit enhanced N400 amplitudes in ERP studies (Chen et al., 2008; Yuan et al., 2011).

The difference waveform (see Figure 3) seems to indicate a sustained effect from P2 time window to late component such as N400, which runs in opposite directions for each group (exclusion/inclusion), which means the typicality effects was task related. The mechanism underlies the difference may be the top-down control of typicality processing. As mentioned in previous context, greater control may lead to lower P2 amplitudes (Chen et al., 2008), also N2 was regarded as an index of cognitive control, more control lead to larger N2 wave (Yeung and Cohen, 2006). Within inclusion group atypical items elicited lower P2 and greater N2 than which of typical members, which indicated atypical items enjoyed more control than typical items in inclusion group while condition reversed for exclusion group. Present task actually composed by two stages, first category, then the inference. For inclusion group, they need to concentrated on within group similarity, and was sensitive to the typicality gradient during the categorization stage, as Kittur et al. (2006) mentioned, atypical items has different features with other category members, so may aroused more control, typicality effect were showed in categorization stage as indexed by P2. But exclusion task was based on the dissimilarity dimension, that is to say during the categorization stage, participants actually need to find out the items which was not a member of the given category, so within group similarity was no so important for categorization, there were no typicality effect found during this stage. However, during the inference stage, items accepted in the classification stage should be rejected in the reasoning stage; category relationship was clearer for typical items than which of atypical items, so more confliction as aroused by typical items as showed by N2 component. The timing analysis confirmed that the inclusion group elicited a typicality effect earlier than did the exclusion group. Combine the control and timing points together; it revealed that inclusion and exclusion aroused different control in the task, the item which conflict to the task requirement may aroused more control. Category typicality played reversed roles in these two tasks, so, lead to the reversed ERPs pattern, typicality processing for inclusion group was earlier than exclusion group.

For N400 component, it is clear that N400 was affected by task, as for Schumacher et al. (2009), in theirs' study, a upward arrow (↑) or a rightward arrow (→) was employed as a cue in a classifying task. If the arrow pointed upwards participants had been instructed to think of a noun that was the category of the preceding word (categorization task);if the arrow pointed rightwards, participants had been instructed to think of a noun that was related to the preceding word, but not of the superordinate category (relation task). In relation task, category word pairs(apple-fruit) and unrelated word pairs elicited the largest N400 wave, but in categorization task, related word pairs (apple-pear) and unrelated word pairs elicited the largest N400 wave (Schumacher et al., 2009). Several researchers has suggested that the N400 amplitude related to prior expectations (van Berkum et al., 1999; Lau et al., 2008). It clearly showed that N400 wave was accorded to the pre-task expectation, items more fit the expectation elicited lower N400 wave. As for inclusion and exclusion group, they hold opposite expectation, inclusion group focused on within group similarity while exclusion group focused on between group dissimilarity, thus, opposite expectation induced the opposite typicality pattern.

In summary, interactions between item type and group were present from as early as the P2 time window to as late as the N400 period. Our ERP data suggest that inclusion and exclusion directly impact categorization and reasoning processes. Inclusion was more sensitive to the typicality gradient than exclusion. The findings presented here build on, but also challenge, current functional interpretations of the N400 component; N400 is not only affected by category relationships, but also by task. Regarding ERPs, interactions between item type and group were shown for P2, N2, and N400 periods.

## Conclusion

In this study, we designed an inclusion and exclusion reasoning task to further examine the behavioral and electrophysiological correlates of the typicality effect. Behavioral data indicated that accuracy and reaction times differed among the three experimental conditions (typical, atypical, and nonmember items). Moreover, the electrophysiological data supported the hypothesis that inclusion and exclusion judgments involve different cognitive processes. Inclusion judgments were more sensitive to category typicality, such that the typicality effect occurred earlier than during exclusion judgments. The typicality effect during inclusion occurred in the P2 and N400 time window, while the exclusion group demonstrated a typicality effect only in the N2 time window. To conclude, inclusion classification is more sensitive to category typicality than is exclusion classification.

## Author contributions

XW propose the experiment and design the procedure, do the most work of the article. HL and YT gave useful comments to the experiment and helped to revise the paper for several times. TT helped to revise the paper for several times and gave useful suggestion about the writing. YX write the E-prime programs; SL helped to analysis data; YT helped to find participants and do the experiment work.

## Funding

This study was supported by the National Natural Science Foundation of China (31100740, 31271088, 31400961, and 30370488) and the MOE Project of Key Research Institute of Humanities and Social Sciences at Universities (11JJD190002).

### Conflict of interest statement

The authors declare that the research was conducted in the absence of any commercial or financial relationships that could be construed as a potential conflict of interest. The Reviewer, JW, and handling Editor declared their shared affiliation, and the handling Editor states that the process nevertheless met the standards of a fair and objective review.
