# Supplementary material for: Categorization Method Affects the Typicality Effect: ERP Evidence from a Category-Inference Task
Source: Front Psychol. 2016 Feb 17;7:184. doi: 10.3389/fpsyg.2016.00184 (PMC4756294; doi:10.3389/fpsyg.2016.00184)
Supplement: Supplementary file 1 [file DataSheet1.docx]

**Appendix:**

**Table 1** Tests of analysis of variance during 170-260 ms

|  | df | SS | MS | F | P | η_p_^2^ |
| --- | --- | --- | --- | --- | --- | --- |
| item type | 2, | 8.817 | 5.499 | 0.156 | 0.809 | 0.005 |
| Error | 60 | 1690.501 | 35.147 |  |  |  |
| frontality | 4 | 1744.727 | 1136.787 | 26.7373 | 0 | 0.471 |
| Error | 120 | 1958.393 | 42.533 |  |  |  |
| Laterality | 2 | 289.269 | 160.784 | 16.847 | 0 | 0.36 |
| Error | 60 | 515.103 | 9.544 |  |  |  |
| Group | 1 | 3271.623 | 3271.623 | 8.273 | 0.007 | 0.216 |
| Error | 30 | 11864.385 | 395.497 |  |  |  |
| item type ×frontality | 8 | 16.665 | 5.677 | 1.592 | 0.198 | 0.05 |
| Error | 240 | 313.960 | 3.565 |  |  |  |
| item type×laterality | 4 | 2.966 | 1.286 | 0.491 | 0.64 | 0.016 |
| Error | 120 | 181.080 | 2.618 |  |  |  |
| item type×group | 2 | 267.824 | 167.049 | 4.753 | 0.019 | 0.137 |
| Error | 60 | 1690.501 | 35.147 |  |  |  |
| frontality ×laterality | 8 | 29.671 | 6.485 | 2.244 | 0.061 | 0.069 |
| Error | 240 | 5893210 | 4.292 |  |  |  |
| frontality×group | 4 | 55.511 | 36.168 | 0.85 | 0.496 | 0.028 |
| Error | 120 | 1958.393 | 42.533 |  |  |  |
| laterality×group | 2 | 25.417 | 14.128 | 1.48 | 0.237 | 0.047 |
| Error | 60 | 515.103 | 9.544 |  |  |  |
| frontality ×laterality×group | 8 | 26.671 | 6.485 | 1.511 | 0.154 | 0.048 |
| Error | 240 | 29.671 | 6.485 |  |  |  |
| item type ×frontality ×laterality | 16 | 3.895 | .518 | 0.608 | 0.761 | 0.02 |
| Error | 480 | 192.163 | .852 |  |  |  |
| item type×frontality×group | 8 | 45.077 | 15.355 | 4.307 | 0.007 | 0.126 |
| Error | 240 | 313.960 | 3.565 |  |  |  |
| item type×laterality×group | 4 | 4.780 | 2.073 | 0.792 | 0.473 | 0.026 |
| Error | 120 | 181.080 | 2.618 |  |  |  |
| item type ×frontality ×laterality×group | 16 | 4.157 | .553 | 0.659 | 0.726 | 0.021 |
| Error | 480 | 192.163 | .852 |  |  |  |

**Table 2** Tests of analysis of variance during the 280-350 ms interval

|  | df | SS | MS | F | P | η_p_^2^ |
| --- | --- | --- | --- | --- | --- | --- |
| item type | 2, | 151.44 | 90.276 | 1.252 | 0.29 | 0.04 |
| Error | 60 | 3629.041 | 72.111 |  |  |  |
| frontality | 4 | 393.112 | 313.83 | 3.737 | 0.052 | 0.111 |
| Error | 120 | 3155.985 | 83.981 |  |  |  |
| Laterality | 2 | 5.336 | 2.699 | 0.18 | 0.833 | 0.006 |
| Error | 60 | 888.253 | 14.975 |  |  |  |
| Group | 1 | 3459.69 | 3459.69 | 5.629 | 0.024 | 0.158 |
| Error | 30 | 18436.962 | 614.565 |  |  |  |
| item type ×frontality | 8 | 65.397 | 25.742 | 4.335 | 0.01 | 0.126 |
| Error | 240 | 452.62 | 76.214 |  |  |  |
| item type×laterality | 4 | 4.525 | 1.407 | 0.425 | 0.749 | 0.014 |
| Error | 120 | 319.123 | 3.307 |  |  |  |
| item type×group | 2 | 636.253 | 379.281 | 5.26 | 0.012 | 0.149 |
| Error | 60 | 3629.041 | 72.111 |  |  |  |
| frontality ×laterality | 8 | 99.707 | 21.844 | 4.101 | 0.002 | 0.12 |
| Error | 240 | 728.865 | 5.323 |  |  |  |
| frontality×group | 4 | 72.442 | 57.832 | 0.68 | 0.443 | 0.022 |
| Error | 120 | 393.112 | 313.83 |  |  |  |
| laterality×group | 2 | 0.046 | 0.023 | 0.002 | 0.998 | 0 |
| Error | 60 | 5.336 | 2.699 |  |  |  |
| frontality ×laterality×group | 8 | 36.309 | 7.955 | 1.494 | 0.201 | 0.047 |
| Error | 240 | 728.865 | 5.323 |  |  |  |
| item type ×frontality ×laterality | 16 | 11.527 | 1.73 | .945 | 0.470 | 0.031 |
| Error | 480 | 365.866 | 1.347 |  |  |  |
| item type×frontality×group | 8 | 52.37 | 20.614 | 3.471 | 0.026 | 0.104 |
| Error | 240 | 452.62 | 76.214 |  |  |  |
| item type×laterality×group | 4 | 9.712 | 3.019 | 2.428 | 0.913 | 0.443 |
| Error | 120 | 319.123 | 3.307 |  |  |  |
| item type ×frontality ×laterality×group | 16 | 5.966 | 0.895 | 0.489 | 0.834 | 0.016 |
| Error | 480 | 365.866 | 1.347 |  |  |  |

**Table 3** Tests of analysis of variance during the 400-500 ms interval

|  | Df | SS | MS | F | P | η_p_^2^ |
| --- | --- | --- | --- | --- | --- | --- |
| item type | 2, | 150.355 | 87.421 | 1.01 | 0.361 | 0.033 |
| Error | 60 | 4464.79 | 86.532 |  |  |  |
| frontality | 4 | 1156.674 | 666.562 | 16.517 | 0 | 0.355 |
| Error | 120 | 2100.887 | 40.356 |  |  |  |
| laterality  Error | 2 | 208.248 | 108.681 | 5.528 | 0.007 | 0.156 |
|  | 60 | 1130.195 | 19.661 |  |  |  |
| Group | 1 | 87.569 | 87.569 | 0.381 | 0.542 | 0.013 |
| Error | 30 | 6889.349 | 229.645 |  |  |  |
| item type ×frontality | 8 | 48.492 | 18.464 | 1.538 | 0.215 | 0.049 |
| Error | 240 | 945.912 | 12.006 |  |  |  |
| item type×laterality | 4 | 31.226 | 10.249 | 2.466 | 0.066 | 0.076 |
| Error | 120 | 379.844 | 4.156 |  |  |  |
| item type×group | 2 | 963.785 | 560.373 | 6.476 | 0.005 | 0.178 |
| Error | 60 | 4464.79 | 86.532 |  |  |  |
| frontality ×laterality | 8 | 21.433 | 4.753 | 0.628 | 0.623 | 0.022 |
| Error | 240 | 942.176 | 6.969 |  |  |  |
| frontality×group | 4 | 383.975 | 22.461 | 0.577 | 0.552 | 0.018 |
| Error | 120 | 2100.887 | 40.356 |  |  |  |
| laterality×group | 2 | 39.776 | 20.758 | 1.056 | 0.352 | 0.034 |
| Error | 60 | 1130.195 | 19.661 |  |  |  |
| frontality ×laterality×group | 8 | 28.416 | 6.302 | 0.094 | 0.473 | 0.029 |
| Error | 240 | 942.176 | 6.969 |  |  |  |
| item type ×frontality ×laterality | 16 | 30.866 | 4.22 | 1.755 | 0.094 | 0.055 |
| Error | 480 | 527.983 | 219.563 |  |  |  |
| item type×frontality×group | 8 | 16.679 | 6.351 | 0.529 | 0.64 | 0.017 |
| Error | 240 | 945.912 | 12.006 |  |  |  |
| item type×laterality×group | 4 | 8.911 | 2951 | 0.71 | 0.55 | 0.023 |
| Error | 120 | 379.844 | 4.156 |  |  |  |
| item type ×frontality ×laterality×group | 16 | 13.405 | 1.832 | 0.762 | 0.626 | 0.025 |
| Error | 480 | 527.983 | 219.563 |  |  |  |
